# An Insight into Enamel Resin Infiltrants with Experimental Compositions

**DOI:** 10.3390/polym14245553

**Published:** 2022-12-19

**Authors:** Claudia Mazzitelli, Uros Josic, Tatjana Maravic, Edoardo Mancuso, Cecilia Goracci, Milena Cadenaro, Annalisa Mazzoni, Lorenzo Breschi

**Affiliations:** 1 Department of Biomedical and Neuromotor Sciences, DIBINEM, University of Bologna, Via San Vitale 59, 40125 Bologna, Italy; 2 Dipartimento di Biotecnologie Mediche, University of Siena, Policlinico Le Scotte, Viale Bracci 1, 53100 Siena, Italy; 3 Department of Medical Sciences, University of Trieste, Strada di Fiume 447, 34125 Trieste, Italy; 4 Institute for Maternal and Child Health-IRCCS “Burlo Garofolo”, Via dell’Istria 65/1, 34137 Trieste, Italy

**Keywords:** resin infiltration, TEGDMA, hydrophobic monomers, solvent, Icon

## Abstract

Resin infiltration is a conservative treatment of initial enamel carious lesions. Only one infiltrant material is available on the market (Icon, DMG), and research is now investigating new chemical compositions so as to further exploit the benefits of the resin infiltration technique. A literature search of the articles testing the effects of different formulations on mechanical properties, resin penetration ability, remineralizing, and antibacterial activities was conducted. Of 238 articles, 29 resulted in being eligible for the literature review. The formulations investigated were all different and consisted in the inclusion of hydrophobic monomers (i.e., BisEMA, UDMA), solvents (ethanol, HEMA), alternative etchants (PAM) or molecules with antibacterial or bioactivity features (i.e., AgNP, YbF_3,_ MTZ, chitosan, DMAMM, HAp, MC-IL, NACP, PUA, CHX) and microfilled resins. Information on the long-term performances of the tested experimental materials were scarce. The combination of TEGDMA with hydrophobic monomers and the inclusion of a solvent alternative to ethanol reinforced mechanical properties of the materials. Hybrid-glass materials demonstrated an enhanced remineralization capacity. Techniques such as tunnelization increased the penetration depth and preserved the recourse to less-conservative treatments. Combining the min-invasive infiltrant approach with remineralizing and bacteriostatic properties would be beneficial for therapeutic and economical aspects, according to the principles of minimally invasive dentistry.

## 1. Introduction

Caries is a chronic disease with the highest incidence worldwide, entailing social and economic implications [1]. An imbalance between demineralization/remineralization processes initiates the carious development through the continuous loss of OH⁻ ions from the apatite crystals of dental enamel. When physiological remineralization fails to compensate for enamel demineralization, translucent, non-cavitated areas of enamel characterized by increased porosity are formed and clinically known as white spot lesions (WSLs). At this stage, preventive protocols can be implemented with individual oral hygiene instructions and diet counseling to avoid bacterial proliferation, which would invariably lead to the formation of cavitated carious lesions. Various treatments can assist with home oral and dietary routines, such as fluoride, xylitol or caseinates [2,3,4]. Despite improvements in the clinical behavior of these materials, in the presence of WSLs, they can only act superficially, as they do not have the ability to reach deep into the demineralized enamel [5]. Moreover, the patient’s cooperation is mandatory to maximize the effects of such treatments.

The use of a resin capable of infiltrating the porosity of demineralized enamel, penetrating, and reaching its depth was first introduced in 2009, with Icon (DMG, Hamburg, Germany) representing the pioneer and, at the moment, the solely patented product present on the market [3,5,6,7]. Icon is a low viscosity, unfilled light-curable solution based on triethylene glycol dimethacrylate (TEGDMA) diluted in ethanol and containing camphoroquinone (CQ) as a photoinitiator. The infiltration technique consists of a first step of hydrochloric acid etching necessary to produce changes in the enamel structure by removing organic material, opening interprismatic spaces and providing mechanical interlocking with the resin [7,8]. In order to more uniformly clean the surface and improve the penetration and retention of the resin, deproteination (i.e., 1% NaOCl) has been recommended before the etching procedures [9,10]. A low viscosity resin is then guided by capillary forces to penetrate deeply into the carious lesion and occlude the dentinal orifices, thus sealing the enamel pores and possibly arresting the caries progression [11,12]. The quality and quantity of resin depth penetration and bond are driven by the chemical composition and mechanical properties of the infiltrant material [12].

Several laboratories [13,14] and clinical studies [12,15] have supported the use of this technique over standard filling practices or resin groove sealants [10,16,17]. Besides providing drill-free treatments, the resin infiltration technique also concurs to decrease tooth sensitivity and improve the esthetics by reducing the pearly white effect typical for the WSLs [18,19,20,21], demonstrating a higher masking effect on labial WSLs when compared to conventional remineralitazion approaches (i.e., hydroxyapatite- and fluoride- based products) [22].

From an esthetic point of view, in the case of mismanipulation or in the case of non-compliance with the instructions for use, the infiltrant resin could be prone to dye absorption possibly jeopardizing the color stability [23,24]. The Icon has undoubtedly allowed for a minimally invasive restorative approach, postponing as much as possible more aggressive treatments thanks to its higher ability to penetrate into the carious lesion compared to sealants or flowable composites [16]. It was noteworthy that some studies observed incomplete resin infiltration of the entire depth of the carious lesion, casting doubts on the tightness of the seal over time [25,26]. However, in those studies, the presence of an organic matrix not completely removed by the sole etching procedures and possibly hampering material’s infiltration, was not taken into consideration [9,10].

Considering the appealing opportunity provided by the resin infiltration technique, the research is continuously moving to expand its therapeutic spectrum by testing new approaches and experimental formulations. Designing a material combining the infiltration mechanism with remineralizing capacity and bacteriostatic activity would be beneficial in further paralyzing the progression of the carious lesion into the underlying dentin [8,27,28,29,30,31]. The technology of the penetrating resins is generating more and more interest among clinicians and researchers, moving toward new formulations that could enhance the strategy established with Icon. In light of these considerations, this literature review aimed to provide a comprehensive perspective of the experimental possibilities currently available to exploit the benefits of the resin infiltrant approach.

## 2. Materials and Methods

Two independent investigators conducted a comprehensive literature review of the 2 databases, Pubmed and Scopus (C.M and T.M.). The following keywords were used: “resin infiltration”, “resin infiltrations”, “resin infiltrant”, “resin infiltrants”, and “experimental”, along with a combination of the different terms. The limit search was set from 2009, when DMG commercialized the first (and unique) infiltrant resin (Icon). Only articles in the English language were taken into consideration. Posters and conference proceedings were not considered for the analysis. The last search was conducted in September 2022. A first selection of the articles was made with the reading of the titles and abstracts. Those studies not considered suitable for inclusion, that is, those articles that did not have the objective of studying experimental formulations of infiltrating resins, were not taken into consideration for the reading of the full text. The following data were extracted from the full-text readings: the author and year of publication, the main country in which the research was conducted (coinciding, where different nationalities are present, to that of the first author), the type of infiltrating resin used (usually taken as a reference for the control group), the objective of the research, experimental formulations of the infiltrating resin, tests performed, and conclusions of the studies. These variables were collected in a custom-made table in MS Excel.

## 3. Results

Of the 238 titles obtained from the first research, those studies not considered relevant to the purpose of the review, i.e., those studies that did not have as their object the infiltrating resins and their experimental formulations, were eliminated. In addition, duplicate articles were also subtracted. A total of 20 articles were considered eligible, with an additional 9 studies included after a cross-sectional analysis of the pertinent bibliography. Finally, 29 articles were considered for inclusion in the review process. The data obtained from the included articles after the review process are presented in Table 1.

The tests were primarily conducted in seven countries, of which Brazil was represented the most [12,30,32,33,34,35,36,37,38,39,40,41,42]. Icon (DMG), the only commercially available infiltrating resin, was the material most used as control [8,12,18,30,31,32,33,35,38,39,40,41,43,44,45,46]. Alternative formulations were created recalling that of the TEGDMA-based Icon product implemented with co-initiators, accelerators and other different molecules depending on the study [8,11,22,29,31,32,34,36,37,47,48,49].

Implementing the bacteriostatic activity of the resin [18,29,30,34,36,37,44,50], improving the remineralization effect [40,43,48,49], and penetration capacity [8,11,12,18,22,28,29,31,38,46,50,51] in the carious lesion, and the reinforcement of the physical-mechanical characteristics [12,33,35,36,39,41,42,47,48,52] of the resins were the main backgrounds of the studies.

The most frequently performed tests concerned the degree of conversion (DC) [12,30,34,36,38,39,40,43,47,49], water sorption/solubility (WS/WS) [35,36,39,49,51], surface micro-hardness of dental tissues [12,30,33,34,35,52], tensile or cohesive strength [32,35,38,39,47], elastic modulus [12,23,33,35,38], contact angle [37,39,49], ion (Ca, P and Fl) release [43,47,49], softening ratio [12,35,47], depth of penetration assessed by light microscopy [44], microradiography [11], confocal microscopy [8,12,28,31,38,42,50], or scanning electron microscopy (SEM) evaluations [22,24,40,43,44,47,52], cytotoxicity [37,43,45,47], antimicrobial activity [29,30,51], and chromatic characteristics [43,48,51].

The majority of the studies focused on the effect of resin infiltration on coronal enamel of permanent teeth [8,11,12,18,29,30,31,33,34,35,36,37,38,39,40,42,43,46,47,48,49], one analyzed the effect of the tunnelization technique on the infiltrant penetration and remineralization of adjacent surface [28], one study investigated the penetration ability of the experimental resin infiltrants with different penetration coefficients and solvents into primary teeth [50], and two studies evaluated the effects of the tested resin infiltrations on root caries lesions [45,46].

The molecules used in the new experimental formulations were: nanomolecules such as nano-Hap [34,35], metrodinazole (MTZ) in combination with Ytterbium trifluoride [18] or 2-(7-methyl-1,6-dioxo-2,5-dioxa-7-octenyl) trimellitic anhydride) (PMMa-n) [44,45], ionic liquid-loaded microcapsules (MC-IL) [37] and ionic salt [36,37,38,39], different nano-particles such as amorphous calcium-phosphate (NACP) [43], silver (AgNP) [28] and spherical zinc (ZnO@NP) [29], polyurethane acrylate oligomer [51], YF_3_ [22,46], chlorhexidine [30,33], dimethylaminododecyl methacrylate (DMADDM) [51], and other bioactive fillers [35,40,49,50,51] and organic fillers [8,31]. Other studies have focused on analyzing different combinations of molecules such as TEGDMA [12,30,33,36,37,38,40,41,42,47,49], BisGMA [11,37,47], BisEMA [12,30,31,32,33,35,36,38,39] and different diluents (none, HEMA, or ethanol). Among the investigated variables, only three studies analyzed the optical characteristics obtained after treatment with the experimental formulations. In two cases, minor color changes were observed when compared to the control group (Icon) [43,51], whilst the inclusion of nano-fluorohydroxyapatite (n-FAHP) resulted in the improved masking effects of artificial WSLs after 14 days [48].

In general, the formulation containing TEGDMA/UDMA positively enhanced the mechanical properties, while the inclusion of ethanol as a diluent resulted in the most deleterious effects. Similarly, pre-heating affected the mechanical properties of the resins investigated [12,30,31,32,33]. Techniques such as tunnelization were able to increase the penetration ability of Icon [28]. The inclusion of organic fillers into Icon formulation demonstrated good penetration ability with enhanced sealing ability, both in interproximal [31] and occlusal [8] cavitated carious lesions with different ICDAS (2,3 and 5, respectively). The remineralization activity could be promoted by the inclusion of hybrid glass with a high resistance to acidic/cariogenic challenges [52].

Only three studies included the aging of the specimens, although with a limited period of time [11,41,51]. No long-term studies were reported, nor were clinical trials present on the use of experimental infiltrant materials with chemical formulations different from Icon.

**Table 1:** Data retrieved from the review analysis. TEGDMA: triethylene glycol dimethacrylate; HEMA: 2-hydroxyethyl methacrylate; PMMAn: 2-(7-methyl-1,6-dioxo-2,5-dioxa-7-octenyl) trimellitic anhydride); MTZ: metronidazole; YbF_3_: Ytterbium trifluoride; DMAEMA: N,N-dimethylaminoethyl methacrylate; CQ: camphoroquinone; UDMA: urethane dimethacrylate; bisEMA: ethoxylated bisphenol A dimethacrylate; EDAB: ethyl 4- dimethylaminebenzoate; DPI: diphenyliodonium hexafluorophosphate salt; DABE: ethyl 4-(dimethylamino)benzoate; PUA: polyurethane acrylate oligomer; FHA: fluorohydroxyapatite particles; FD-BG: fluoride-doped bioactive glass; YF_3_: Ytterbium-fluoride; CA: Chloridric acid; PA: Phosphoric acid; PAM: phosphoric acid 2-hydroxyethyl methacrylate este; DMADDM: dimethylaminododecyl methacrylate; ZnO@NP: Zinc nanoparticles. BAG: Bioactive glass; n-FHAP: nano-fluorohydroxyapatite.

Author (Year)	Country	Resin Infiltrant	Objective of the Study	Alternative Formulations	Test	Conclusions
Andrade Neto et al. (2016) [34]	Brazil	⚬ Control: light-curable resin blend composed of 0 wt% UDMA, 87 wt% TEGDMA, 0.5 wt% CQ, 1 wt% ethyl 4-dimethylaminebenzoate (coinitiator) and 1.5 wt% diphenyliodonium hexafluorophosphate; ⚬ Control + HAp0 (no hydrothermal treatment (HT)); ⚬ Control + HAp2 (2 h of HT); ⚬ Control + HAp5 (5 h of HT).	To evaluate the influence on DC and anti-caries efficacy	10%wt HAp after HT	XRD, FT-IR, DC, TEM, Knoop hardness	10%wt HAp after 2 or 5 h of HT were equally effective in increasing DC and enhancing anti-caries capacity
Araujo et al. (2015) [32]	Brazil	⚬ 100 wt% TEGDMA; ⚬ 80 wt% TEGDMA, 20 wt% ethanol; ⚬ 80 wt% TEGDMA, 20 wt% HEMA; ⚬ 75 wt% TEGDMA, 25 wt% UDMA; ⚬ 60 wt% TEGDMA, 20 wt% UDMA, 20 wt% ethanol; ⚬ 60 wt% TEGDMA, 20 wt% UDMA, 20 wt% HEMA; ⚬ 75 wt% TEGDMA, 25 wt% BisEMA; ⚬ 60 wt% TEGDMA, 20 wt% BisEMA, 20 wt% ethanol; ⚬ 60 wt% TEGDMA, 20 wt% BisEMA, 20 wt% HEMA.	To evaluate the influence on bond strength	Different monomer (UDMA and BisEMA) and solvent (ethanol and HEMA)	Microtensile bond strength test	The resin matrix did not affect bond strength. Ethanol negatively influenced the bond strength, possibly interefering with the polymerization reaction.
Araujo GSA et al. (2013) [12]	Brazil	⚬ Control: Icon; ⚬ 100 wt% TEGDMA; ⚬ 80 wt% TEGDMA, 20 wt% ethanol, ⚬ 100 wt% TEGDMA, 20 wt% HEMA; ⚬ 75 wt% TEGDMA, 25 wt% BisEMA; ⚬ 60 wt% TEGDMA, 20 wt% BisEMA, 20 wt% ethanol; ⚬ 60 wt% TEGDMA, 20 wt% BisEMA, 20 wt% HEMA; ⚬ 75 wt% TEGDMA, 25 wt% UDMA; ⚬ 60 wt% TEGDMA, 20 wt% UDMA, 20 wt% ethanol; ⚬ 60 wt% TEGDMA, 20 wt% UDMA, 20 wt% HEMA	To evaluate the influence of monomers and solvents on mechanical properties and penetrativity	UDMA, BisEMA, HEMA, ethanol	DC, Knoop hardness, softening ratio, E- modulus, confocal laser scanning microscope	Hydrophobic monomers and solvents, in particular ethanol, resulted in decreased mechanical properties and did not improve penetrativity.
Askar et al. (2018) [31]	Germany	⚬ Icon (control); ⚬ 55 wt% Icon + 45 wt% organic fillers (MFIR); ⚬ Icon (3 min) + flowable composite (IR+FC).	Penetration/filling ability in non-, micro- and cavitated inter-proximal natural caries lesions (ICDAS 2, 3, and 5).	Organic filler particles	Dual-fluorescence staining and confocal microscopy	MFIR showed similar penetration depth as IR with improved filling ability.
Bagheri et al. (2020) [48]	Iran	⚬ 100% TEGDMA; ⚬ 98 wt% TEGDMA + 2 wt% n-FHAP; ⚬ 95 wt% TEGDMA + 5 wt% n-FHAP; ⚬ 98 wt% TEGDMA + 2 wt% BAG; ⚬ 95 wt% TEGDMA + 5 wt% BAG.	To evaluate the masking effects of artificial WSLs	BAG and n-FHAP	Spectrophotometry	n-FHAP-based compositions showed good masking effects after 14 days.
Cuppini et al. (2021) [37]	Brazil	⚬ Control: 90 wt% TEGDMA, 10 wt% Bis-GMA, 1 mol% CQ and ethyl 4-dimethylaminobenzoate ⚬ Control + 2.5 wt% MC-IL; ⚬ Control + 5 wt% MC-IL; ⚬ Control + 10 wt% MC-IL.	To evaluate the influence on mechanical properties and citotoxicity	Ionic liquid-loaded microcapsules (MC-IL)	SEM, ultimate tensile strength, contact angle, and surfaces free energy	MC-IL increased surface properties without influencing mechanical properties and cytotoxicity.
Dai et al. (2022) [43]	China	⚬ Control: Icon; ⚬ 90 wt% Icon + 10 wt% NACP; ⚬ 80 wt% Icon + 20 wt% NACP; ⚬ 70 wt% Icon + 30 wt% NACP; ⚬ 60 wt% Icon + 40 wt% NACP	To evaluate the remineralization ability and mechanical properties	Amorphous calium-phosphate nanoparticles (NACP)	Citotoxicity, DC, microhardness, color change, Ca and P ion release, and pH cycling.	Incorporating of 30 wt% NACP resulted in longer Ca and P release, higher DC, higher hardness, and biocompatibility
Fischer et al. (2021a) [44]	Poland	⚬ Control: Icon; ⚬ 75 wt% TEGDMA; 25 wt% HEMA; 1 wt% PMMAn-MTZ; 1 wt% DMAEMA; 0.5 wt% CQ.	To evaluate the depth penetration into root cementum	PMMAn-MTZ	SEM and light microscopy	Experimental resin showed good penetration into decalcified root and in the root dentin.
Fischer et al. (2021b) [45]	Poland	⚬ Control: Icon; ⚬ 75 wt% TEGDMA, 25 wt% HEMA, 1 wt% PMMMn-MTZ, 1 wt% DMAEMA, 0.5 wt% CQ.	To evaluate the influence on citotoxicity	PMMMn-MTZ	Mouse subcuta- neous connective tissue fibroblasts, fluorescence microscope, and viability assessment	No differences in cytotoxicity among the tested groups.
Flor-Ribeiro et al. (2019) [36]	Brazil	⚬ Control: 75 wt% TEGDMA; 25 wt% BisEMA; 10 wt% HEMA; 0.5 wt% CQ; 1 wt% EDAB. ⚬ Control + 0.12 wt% chitosan; ⚬ Control + 0.25 wt% chitosan; ⚬ Control + 0.5 mol% DPI; ⚬ Control + 0.5 mol% DPI + 0.12 wt% chitosan; ⚬ Control + 1 mol% DPI; ⚬ Control + 1 mol% DPI + 0.25 wt% chitosan; ⚬ Control + 1 wt% DPI + 0.12 wt% chitosan; ⚬ Control + 1 mol% DPI + 0.25 wt% chitosan.	To evaluate the influence on mechanical properties and bacteriostaticity	Ionic salt (DPI) and chitosan	DC, E-modulus, flexural strength, water sorption/solubility, and antibacterial ability.	The concentration of 0.5 mol% DPI and 0.12 wt% chitosan could improve mechanical properties and enhance antibacterial activity in the experimental infiltrant resin.
Gaglianone et al. (2020) [38]	Brazil	⚬ Icon; ⚬ INF: 75 wt% TEGDMA; 25 wt% Bis EMA; 0.5 wt% CQ; 1 wt% DABE; ⚬ INF-E: 65 wt% TEGDMA; 25 wt% BisEMA; 10 wt% ethanol; 0.5 wt% CQ; 1 wt% DABE. ⚬ INF-H: 65 wt% TEGDMA; 25 wt% BisEMA; 10 wt% HEMA; 0.5 wt% CQ; 1 wt% DABE; ⚬ TEG: 100 wt% TEGDMA; 0.5 wt% CQ; 1 wt% DABE; ⚬ TEG-E: 90 wt% TEGDMA; 10 wt% ethanol; 0.5 wt% CQ; 1 wt% DABE; ⚬ TEG-H: 90 wt% TEGDMA; 10 wt% HEMA; 0.5 wt% CQ; 1 wt% DABE.	To evaluate the influence on physical properties and penetration depth.	Pre-heating (25 and 55 °C) and different formulation with HEMA or ethanol as solvents.	DC, E-modulus, flexural strength, contact angle, confocal microscopy	Pre-heating and the adding of ethanol as solvent resulted in altered mechanical properties and did not enhance penetration depth.
Hashemian et al. (2021) [49]	Iran	⚬ Control: 98 wt% TEGDMA; 0.5 wt% CQ; 1.5 wt% DABE; ⚬ 10 wt% PUA; 88 wt% TEGDMA; 0.5 wt% CQ; 1.5 wt% DABE.	To improve the physical properties and enhance remineralization	PUA, nano-FHA and FD-BG fillers	CA, penetration coefficient, field emission SEM, EDS	The combination of PUA and TEGDMA may improve mechanical properties and inclusion of FD-BG may include reminerilizing effects.
Inagaki et al. (2016a) [30]	Brazil	⚬ Control: Icon; ⚬ 100 wt% TEGDMA; ⚬ 99.9 wt% TEGDMA, 0.1 wt% CHX; ⚬ 99.8 wt% TEGDMA, 0.2 wt% CHX; ⚬ 75 wt% TEGDMA, 25 wt% UDMA; ⚬ 74.9 wt% TEGDMA, 25 wt% UDMA, 0.1 wt% CHX; ⚬ 74.8 wt% TEGDMA, 25 wt% UDMA, 0.2 wt% CHX; ⚬ 75 wt% TEGDMA, 25 wt% BisEMA; ⚬ 74.9 wt% TEGDMA, 25 wt% BisEMA, 0.1 wt% CHX; ⚬ 74.8 wt% TEGDMA, 25 wt% BisEMA, 0.2 wt% CHX.	To evaluate the influence of mechanical properties and bacteriostaticity.	CHX and different hydrophobic monomers.	DC, Knoop hardness, microbiological assay.	TEGDMA/UDMA/CHX had improved antimicrobiological properties among groups without influencing mechanical properties.
Inagaki et al. (2016b) [33]	Brazil	⚬ The same as in “Tiemi Inagaki et al., 2016a”	To evaluate the influence on chemio-physical properties	CHX and different hydrophobic monomers.	Sorption and solubility, softening ratio, Flexural strength, and E-modulus.	The monomers’ characteristics more relevantly influence the physio-chemical characteristics of the resin than the inclusion of CHX. The combination of TEGDMA/UDMA resulted in higher properties.
Kielbassa et al. (2020) [28]	Austria	⚬ Internal infiltration: Icon + light-curing adhesive (G Premio Bond, GC Europe, Leuven, Belgium) and G-aenial FlowX (GC Europe); ⚬ External infiltration: Control (Icon); Experimental (Icon + 20 nm AgNP)	To evaluate penetrativity of internal and external infiltration	AgNP	Confocal laser scanning microscope	AgNP did not influence the penetrativity of Icon. Internal and external infiltration was considered a viable operative chance to treat proximal lesions extending to dentin.
Lausch et al. (2017) [8]	Germany	⚬ Icon (control); ⚬ 45% methacrylate based pre-polymerized filler and 55% Icon, MFIR; ⚬ Icon for 3 min + fissure sealant (Helioseal, Ivoclar Vivadent), ISC.	Penetration/filling ability in occlusal natural caries lesions (ICDAS 2, 3 and 5).	Organic filler particles	Dual-fluorescence staining and confocal microscopy	MFIR could combine the penetration depth of RI + the sealing ability of a fissure sealant.
Mathias et al. (2019) [39]	Brazil	⚬ Control: Icon; ⚬ Monomeric experimental base: 25 wt% BisEMA, 75 wt% TEGDMA, 0.5 mol% CQ, 1 mol% EDAB; ⚬ DPI at different concentrations (0.25 Mol%, 0.5 mol%, 1 mol%); ⚬ Experimental solvents (10 wt% HEMA or 10 wt% ethanol).	To evaluate the influence on mechanical properties	DPI and different solvents (HEMA or ethanol)	Degree of conversion, water sorption/solubility, cohesive strength, contact angle.	0.5 mol% DPI may compensate some worsening in mechanical properties in HEMA and ethanol-diluted resin infiltrants. Ethanol worsened the properties tested.
Nedeljkovic et al. (2022) [52]	Holland	⚬ No treatment (control); ⚬ Icon; ⚬ Icon + 1 wt% HaP; ⚬ Hybrid-glass monomer/oligomer (15%); ethanol.	Remineralization after artificial cariogenic challenges.	Hybrid-glass monomer/oligomers	Knoop microhardness, surface roughness SEM	Hybrid glass monomers performed better than control thanks to its acid/cariogenic higher stability.
Nóbrega et al. (2020) [41]	Brazil	⚬ Control: Icon; ⚬ 60 wt% TEGDMA, 20 wt% UDMA, 20 wt% HEMA; ⚬ 80 wt% TEGDMA, 20 wt% HEMA; ⚬ 75 wt% TEGDMA, 25 wt% BisEMA.	To evaluate the influence on mechanical properties	Different composition and different enamel carious layers after 21 days	Microhardness and fluorescence microscopy	The combination TEGDMA/ BisEMA was the less affected by hydrolytic degradation.
Nowak-Wachol et al. (2022) [46]	Poland	⚬ Control: Icon ⚬ 0.05 gr PMMAn-MTZ; 3.75 g TEGDMA; 1.25 g HEMA 0.5% CQ 1%DMAEMA ⚬ With 2% or 4% YF_3_.	To evaluate the penetrativity into decalcified root cementum	YF_3_	SEM/EDX	4% YF_3_ hampered penetrativity, while no differences were observed with 2% YF_3_
Obeid et al. (2022) [40]	Brazil	⚬ Control: Icon; ⚬ Icon + nanofibers filled with SiO_2;_ ⚬ Icon + nanofibers filled with SiO_2_-CaP	To increase mechanical properties and enhance anticaries effect	Bioactive hybrid nanofibers containing silica (SiO2) or calcium-doped silica (SiO2–CaP)	DC, Knoop hardness, EDX	Bioactive nanofibers resulted in increased SH, with Si-O_2_CaP enabling higher remineralizing potential.
Paris and Meyer-Lueckel (2010) [11]	Germany	⚬ 24.75 wt% BisGMA, 74.25 wt% TEGDMA, 0.5 wt% CQ, 0.5 wt5 DABE; ⚬ 19.8 wt% BisGMA, 59.4 wt% TEGDMA, 19.8 wt% ethanol, 0.5 wt% CQ, 0.5 wt% DABE; ⚬ 99 wt5 TEGDMA, 0.5 wt% CQ, 0.5 wt% DABE; ⚬ 79.2 wt% TEGDMA, 19,8 wt% ethanol, 0.5 wt% CQ, 0.5 wt% DABE.	To evaluate the penetration ability	Infiltrant materials with different penetration coefficient	Microradiography after 400 days in demineralizing environment	TEGDMA showed higher penetration ability. Supposedly, ethanol contrasted with the polymerization reaction.
Paris et al. (2012) [50]	Germany	⚬ The same as in “Paris and Meyer-Lueckel (2010)	To evaluate the penetration ability in primary teeth	Infiltrant materials with different penetration coefficient and solvents in primary teeth	Confocal laser scanner microscopy	The penetration coefficient did not influence the penetration ability of the experimental resins in primary teeth. The solvent was a detrimental factor.
Prodan et al. (2022) [47]	Romania	⚬ 50 wt% TEGDMA, 20 wt% HEMA, 30 wt% UDMA, BaF2 ⚬ 60 wt% TEGDMA, 20 wt% HEMA, 20 wt% BisGMA, BaF2 ⚬ 70 wt% TEGDMA, 30 wt% UDMA, BaF2 ⚬ 55 wt% TEGDMA, 30 wt% UDMA, 15 wt% ethanol, BaF2 ⚬ 75 wt% TEGDMA, 25 wt% HEMA, BaF2 ⚬ 60 wt% TEGDMA, 20 wt% HEMA, 20 wt% BisGMA, BaF2 ⚬ Control: Icon.	To evaluate the influence on mechanical properties	Different monomer mixtures with BaF2	DC, Water sorption, SEM, Fl release, flexural strength, Young’s modulus, residual monomers	Best mixture was found in Bis-GMA/HEMA/ TEGDMA
Sfalcin et al. (2017) [35]	Brazil	⚬ Control: Icon; ⚬ Filler-free control (CR): 74 wt% TEGDMA; 24.5 wt% BisEMA; 0.5% CQ; 1 wt% EDAB. ⚬ CR + 10 wt% HAp; ⚬ CR + 10 wt% amorphous calcium phosphate; ⚬ CR + 10 wt% bioactive glass Zn; ⚬ CR + 10 wt% bioactive glass 45S5; ⚬ CR + 10 wt% alcium silicate microfillers modified with β-TCP.	To evaluate the influence on mechanical properties	Bioactive fillers	FT-IR, universal testing machine, DC, Knoop hardness, softening ratio, tensile cohesive strength, E-modulus, water sorption/solubility.	Bioactive fillers increased chemo-mechanical properties of experimental resins compared to Icon, except for softening ratio.
Skucha-Nowak et al. (2016) [18]	Poland	⚬ Control: Icon + YbF_3_; ⚬ Experimental: TEGDMA, HEMA, PMMAn-MTZ, YbF_3,_ DMAEMA, CQ	To improve penetrativity and bacteriostaticity	MTZ + YbF_3_	Back-scattered SEM	No improvement in penetration depth (probably due to the grain size of YbF_3_). No information on bacteriostaticity after inclusion of MTZ.
Villegas et al. (2020) [29]	Argentina	⚬ Control: Icon; ⚬ Control + 2 mg/mL ZnO@NP	To add antibacterial activity	ZnO@NP	Microdilution method, infiltration, SEM/EDS	Experimental group showed antibacterial activity and better infiltration than the control.
Wang et al. (2021) [42]	Brazil	⚬ Control: Icon + CA; ⚬ Icon + PA; ⚬ Icon (no CA); ⚬ TEGDMA + PA; ⚬ TEGDMA ⚬ 75 wt% TEGDMA + 25 wt% PAM; ⚬ 75 wt% TEGDMA + 25 wt% PAM + PA; ⚬ 50 wt% TEGDMA + 50 wt% PAM; ⚬ 50 wt% TEGDMA + 50 wt% PAM + PA; ⚬ 25 wt% TEGDMA + 75 wt% PAM; ⚬ 25 wt% TEGDMA + 75 wt% PAM + PA; ⚬ PAM + PA; ⚬ PA.	To evaluate the influence on mechanical properties and penetration depth	Self-etching capacity with PAM	Confocal laser scanning microscope, viscosity, pH.	Icon and CA demonstrated the lower penetrativity, while this was improved in the formulation TEGDMA/PAM
Yu et al. (2020) [51]	China	⚬ Control: Icon; ⚬ Icon + 2.5% DMADDM; ⚬ Icon + 5% DMADDM; ⚬ Icon + 10% DMADDM.	To add antibacterial activity	DMADDM	Citotoxicity, AFM, colour, biofilm formation,	Experimental resins showed good antibacterial activity after 1 month and did not influence the surface roughness and colour stability.

## 4. Discussion

The benefits of resin infiltration for the treatment of cavitated and non-cavitated carious lesions, both on interproximal and occlusal dental tissue, have been widely accepted [17,32,50,52,53].

From a chemical point of view, Icon is a low viscosity, unfilled light-curable (CQ) solution based on TEGDMA diluted in ethanol. The absence of fillers favors its very low viscosity, giving it the ability to penetrate deeper spaces than a more viscous resin [10,28,31,50]. The prevalence of TEGDMA in Icon composition, considered a highly hydrophobic molecule par excellence, influences the interaction of the resin with the enamel tissue [35,50]. The presence of ethanol has been considered the most significant limitation in resin infiltrant’s composition, as it may hamper the penetration of the resin up to the entire depth of the carious lesion [39] and might not be completely evaporated, possibly compromising the polymerization reaction [47,54]. In light of the aforementioned considerations, researchers continuously move to expand the possible use of Icon by investigating alternative resin formulations to prevent caries progression, postpone aggressive treatments and, finally, fulfill the minimum intervention dentistry requirements [27,28,49].

When formulating novel molecules, it is fundamental to consider their physical–mechanical characteristics, which must correspond to well-defined physical and biological requirements [55]. The new-designed infiltrant materials are requested to protect the infiltrated tissue and the adjacent demineralized area from recurrent bacterial attacks, thus preventing caries progression. To this end, an ideal resin infiltrant material should possess specific mechanical properties to interact with the demineralized/decalcified tooth tissues, withstand occlusal forces or functional habits (i.e., brushing) and prolong the effectiveness of the seal over time (Table 2). Even though this concept could be hard to obtain in a single product, nanotechnology, and the possibility of introducing bioactive fillers into the material’s composition, it represents feasible strategies to fulfill the requirements of minimally invasive dentistry and impart bioactivity.

### 4.1. Mechanical Properties

The mechanical properties and physical-chemical characteristics regulate the therapeutic effects of the material. Accordingly, any improvement in the material’s properties would result in an increased therapeutic effect.

The degree of conversion (DC) of a material assumes importance not only for the degree of loose carbon–carbon bonds, but also because it, in turn, conditions many parameters such as water sorption and solubility, surface hardness, color stability, and chemo-mechanical properties [56]. The basic characteristic that an infiltrating resin must possess is a DC of not less than 50%. The DC of Icon was estimated around 55% [43,57], eventually limited by the high concentration of TEGDMA, which is characterized by a rapid vitrification effect that could limit the polymeric conversion of the resin [58]. Some authors have speculated that the high amount of TEGDMA and the absence of filler contents in Icon formulations could affect the resin micro-hardness [35,59], as well as its high molecular weight might influence the solubility rate of the resin [60].

The DC is directly related to the water sorption of a resin-based material, according to which the lower the DC, the higher the water sorption [61]. The water sorption/solubility regulates several physio-chemical mechanisms, possibly negatively influencing the structure of the material. A degraded polymer matrix would reduce the longevity and function of resin-based materials [54,62,63,64].

TEGDMA, the main component of Icon, is a highly hydrophilic monomer, possibly exposing the resin to premature hydrolytic degradation [28,65]. The inclusion in Icon of additional monomers (i.e., hydrophobic) could enhance the material’s mechanical properties and maximize its therapeutical potential [58]. Accordingly, more hydrophobic, low-viscosity monomers, i.e., UDMA and BisEMA, have been tested [12,30,32,47]. Studies have demonstrated that the inclusion of such hydrophobic monomers into the resin infiltrant Icon did not impair its mechanical properties but could be proposed as an enhanced formulation [32]. Among these chemical combinations, TEGDMA/UDMA resulted more than others in improved DC, E-modulus, surface hardness, and bond strength [12,30,32].

Previously, an increase in enamel surface hardness after using Icon as infiltrating resin has been observed [66]. The experimental formulations have demonstrated a beneficial effect in increasing the surface hardness of the enamel with which the resin comes into contact. This feature becomes even more necessary in the case of a tooth affected by developmental defects (i.e., MIH, amelogenesis imperfecta, etc.) [67]. In those teeth, the resin has demonstrated better effects than resinous sealants when used in cavities with ICDAS 1 and 2 [68,69]. Since the teeth with developmental defects are highly exposed to premature fracture [4], increasing the surface hardness would allow them to increase the possibilities of maintaining the dental structure over time.

The type of solvent has been considered the most influencing factor on the mechanical properties of experimental resin infiltrants [12,32,38,39]. In particular, the presence of ethanol in their composition could impair the mechanical properties, possibly due to an interference with the polymerization of the material resulting in reduced cohesive strength, increased softening, higher water sorption, and contact angle [12,32,39], and finally influencing the depth of penetration of the resin [12].

### 4.2. Penetration Depth

A penetration coefficient (PC) equal to or higher than 100 Cm/s is essential to achieve a uniform penetration into WSLs [12,49]. Materials mainly composed of TEGDMA with PC higher than 200 Cm/s demonstrated a higher penetration ability and the chance to inhibit the progression of the carious lesion with respect to other materials in which more hydrophobic monomers and solvents have been added [11]. However, when the same resin formulations were used on primary teeth, no differences in penetration depth were observed between resins with different penetration coefficients [50]. Notwithstanding these results on primary teeth, as previously affirmed for permanent teeth [11], resins with higher penetration coefficient and ethanol-free should be preferred in order to achieve deeper resin infiltration [11,50].

The penetration depth is directly correlated with the ability to arrest the caries progression [7,14,70]. Ideally, an infiltrant resin should possess a high PC in a clinically reasonable application time [11]. Taking into account the first aim of infiltration therapy able to postpone less conservative restorative procedures, re-application of the resin into already infiltrated areas has been proposed to reinforce the beneficial effects of Icon [10,28]. Moreover, the inclusion of micro-organic fillers into Icon composition could provide a better sealing and contribute to prolonging the durability of the resin infiltrant over time [31]. Regarding the alternative formulations found in the present literature review, many attempts made to increase resin penetration capacity consisted in the inclusion of monomer mixtures such as BisGMA, HEMA, or BisEMA. According to the results, the monomer combination often resulted in the worsening of the material’s mechanical properties and did not always improve penetration depth. In order not to interfere with viscosity and penetration, the filler concentration should not exceed the 15%wt [34,71]. This should be taken into consideration when formulating novel resin infiltrant formulations. Alternative techniques have been proposed to overcome the problem of the incomplete penetration depth, such as double internal/external infiltration [10,28] or resin pre-heating [38]. The internal/external infiltration has been considered a viable technique to treat proximal lesions extending to dentin [10,28], contributing to the occlusion and stabilization of caries lesion porous areas and resulting in the preservation of the proximal contact area [10]. This technique should be further explored to increase the lifetime of the infiltrating material. Instead, resin pre-heating, in combination with alternative solvents, did not improve mechanical properties nor increased the penetrability into the enamel [38]. The solvent should increase the PC of the material into the enamel rods; however, structural inhomogeneities in the form of unpolymerized areas within the resin attributing to the type of solvent, of which ethanol was considered the major culprit, have been observed [11,32,38].

### 4.3. Antibacterial Activity

Different molecules with previously proved bacteriostatic capacity have been included in the original formulation of Icon to evaluate their effects on the mechanical properties and assess their validity as antibacterial products [18,21,28,30,33,34,35,36,37,39,40,44,51]. The review has highlighted a heterogeneity of molecules tested, and it was challenging to outline which is the most performing formulation for the purpose of antibacterial activity. All investigated experimental formulations resulted in enhanced antibacterial activity in a short period of time. However, these results need to be confirmed by long-term studies.

### 4.4. Remineralization Activity

The combination of a microinvasive infiltrant approach with remineralization capacity would represent a therapeutic possibility to postpone restorative procedures over time, with consequent cost savings for both patients and health institutions [72]. Several strategies have been proposed to furnish Icon with certain bioactivity by blending bioactive fillers and fluoride complexes into the resin composition. The formation of fluorapatite complexes has been confirmed to increase the resistance of dental tissues to acid attacks [56]. The inclusion of fluoride-containing complexes in composite resins and orthodontic cements has demonstrated promising results in preventing the formation of WSLs and reducing bacterial biofilm rooting. Hashemian et al. evaluated the effects of remineralizing fillers such as fluorohydroxyapatite (FHA) and fluoride-doped bioactive glass (FD-BG) added to an experimental TEGDMA-based infiltrating resin [49]. According to the authors, promising remineralizing results have been achieved with the experimental formulations without affecting the resin’s mechanical properties. Very recently, Dai et al. have evaluated the re-demineralizing ability of nanoparticles of amorphous calcium phosphate (NACP)-based compound [43]. The incorporation of 30% NACP into the Icon formulation resulted in increased mechanical properties, optimal biocompatibility, and the possibility of Ca and P ion release over a period of 14 days [43]. Many factors may influence the material’s bioactivity in the oral cavity, such as the saliva and patient’s compliance [73], and these should be considered in future studies.

### 4.5. Color Stability

WSLs represent the first manifestation of an initial, non-cavitated carious lesion. The initial subsurface demineralization promoted by a bacterial acidic attack leads to the formation of micropores between enamel rods, mineral loss, and increased surface roughness. From an optical point of view, this phenomenon results in light refractive alterations creating the typical pearly effect of [74]. WSLs represent a critical esthetical issue, often creating discomfort and consequences in the social life of adolescents and adults. The resin infiltration technique ameliorates the white appearance by filling the spaces between enamel rods [3,51]. One study experimented different combinations of TEGDMA with bioactive glass or nano-fluorohydroxyapatite. Irrespective of the percentage, the latter obtained good masking effects of artificial WSLs maintained after 14 days [48]. Among the studies analyzed, only two focused on the effect of Icon and the experimental formulation on in vitro enamel optical properties [43,51]. Neither the quaternary ammonium compound [51] nor the calcium phosphate nanoparticles [43] investigated altered the chameleon performance of resin infiltration, presuming the effectiveness of these formulations from an esthetical point of view. Considering the corroborated results on the antibacterial activity of quaternary ammonium compounds [75] and the remineralizing effects of CaP nanoparticles [24], it would be worthwhile to push additional investigations in their direction.

### 4.6. Root Caries

Considering the lengthening of the average life span and the increasing possibility of preserving many teeth in the oral cavity, geriatric dentistry is becoming increasingly popular. Dental tissues undergo a number of physiological changes over time, of which gingival retraction, root exposure, and the possibility of developing non-carious cervical lesions (NCCLs) are the primary consequences of the aging process [76,77]. In the presence of non-optimal hygiene and pathological situations that occur with age (diabetes, drug intake, etc.), the patient is easily exposed to the development of caries. In this case, a minimally invasive therapeutic intervention, such as the resin infiltration technique, could be an optimal clinical option in the case of a debilitated or poorly cooperative elderly patient.

Taking these aspects into consideration, the effect of several experimental infiltrating resins has also been tested on cement and root dentin [45,46]. A pilot study suggested the possible use of an experimental resin composed of a mixture of TEGDMA, HEMA, DMAEMA and antibacterial components able to penetrate root tissues, not only enamel/cementum but also at the root/dentin level [45]. Contrarily, no differences between experimental blends and commercial solutions were identified when YF3 was included to increase the coefficient penetration into root caries [46].

## 5. Conclusions

The resin infiltration technique, with its pioneer product Icon, has expanded the dogma of minimally invasive dentistry. Great efforts are continuously made in the material’s technology to provide a material with increased potentials. Within the limits of this study, the combination of TEGDMA and hydrophobic monomers (i.e., TEGDMA/UDMA), other solvents alternative to ethanol, and molecules with antibacterial capacities seems to be the direction to which research is directed. Techniques such as tunnelization or the inclusion of microfiller or hybrid glasses resulted in improved penetration depth, sealing ability, and remineralizing effects. However, the heterogeneity of the experimental compositions present in the literature does not allow for a standardization of the formulation.

## Figures and Tables

**Table 2:** Template summarizing an ideal resin infiltrant material according to the information found in the literature regarding formulations and techniques. This literature review helped unfold limits and possibilities of the experimental formulations. It was not possible to extrapolate clear indication regarding the best materials to increase the antibacterial capacity and color stability of a potential novel resin infiltrant due to a variety of molecules tested with no clear standardization of formulations and setup of the studies.

	Composition		Characteristics					
	Monomer	Solvent	Penetration Depth	Sealing	Antibacterial Capacity	Remineralization Ability	Color	Root Caries
Ideal Resin Infiltrant	Hydrophobic monomer (UDMA)	HEMA	Re-application into already infiltrated areas or internal/external infiltration (tunnelization)	Micro-organic fillers	No clear indication	Nano-particles (fluoride or NACP)	No clear indication	TEGDMA/HEMA/ DMAEMA

## Data Availability

Not applicable.
